# Human mitochondrial pyruvate carrier 2 as an autonomous membrane transporter

**DOI:** 10.1038/s41598-018-21740-z

**Published:** 2018-02-22

**Authors:** Raghavendra Sashi Krishna Nagampalli, José Edwin Neciosup Quesñay, Douglas Adamoski, Zeyaul Islam, James Birch, Heitor Gobbi Sebinelli, Richard Marcel Bruno Moreira Girard, Carolline Fernanda Rodrigues Ascenção, Angela Maria Fala, Bianca Alves Pauletti, Sílvio Roberto Consonni, Juliana Ferreira de Oliveira, Amanda Cristina Teixeira Silva, Kleber Gomes Franchini, Adriana Franco Paes Leme, Ariel Mariano Silber, Pietro Ciancaglini, Isabel Moraes, Sandra Martha Gomes Dias, Andre Luis Berteli Ambrosio

**Affiliations:** 1 Laboratório Nacional de Biociências, Centro Nacional de Pesquisa em Energia e Materiais, Campinas, SP 13083-970 Brazil; 2 Membrane Protein Laboratory, Diamond Light Source, Harwell Science and Innovation Campus, Didcot, Oxfordshire OX11 0DE England; 3 Research Complex at Harwell, Rutherford Appleton Laboratory, Harwell, Didcot, Oxfordshire OX11 0FA England; 4 Departamento de Química, Faculdade de Filosofia, Ciências e Letras de Ribeirão Preto, Universidade de São Paulo, Ribeirão Preto, SP, 14040-901 Brazil; 5 Laboratory of Biochemistry of Tryps – LaBTryps, Departamento de Parasitologia, Instituto de Ciências Biomédicas, Universidade de São Paulo, São Paulo, SP 05508-000 Brazil; 6 Present Address: Structural Genomics Consortium (SGC), Universidade Estadual de Campinas, Campinas, SP 13083-886 Brazil; 7 Present Address: Departamento de Bioquímica e Biologia Tecidual, Instituto de Biologia, Universidade Estadual de Campinas, Campinas, SP 13083-862 Brazil; 8 Present Address: National Physical Laboratory, Teddington, Middlesex TW11 0LW England

## Abstract

The active transport of glycolytic pyruvate across the inner mitochondrial membrane is thought to involve two mitochondrial pyruvate carrier subunits, MPC1 and MPC2, assembled as a 150 kDa heterotypic oligomer. Here, the recombinant production of human MPC through a co-expression strategy is first described; however, substantial complex formation was not observed, and predominantly individual subunits were purified. In contrast to MPC1, which co-purifies with a host chaperone, we demonstrated that MPC2 homo-oligomers promote efficient pyruvate transport into proteoliposomes. The derived functional requirements and kinetic features of MPC2 resemble those previously demonstrated for MPC in the literature. Distinctly, chemical inhibition of transport is observed only for a thiazolidinedione derivative. The autonomous transport role for MPC2 is validated in cells when the ectopic expression of human MPC2 in yeast lacking endogenous MPC stimulated growth and increased oxygen consumption. Multiple oligomeric species of MPC2 across mitochondrial isolates, purified protein and artificial lipid bilayers suggest functional high-order complexes. Significant changes in the secondary structure content of MPC2, as probed by synchrotron radiation circular dichroism, further supports the interaction between the protein and ligands. Our results provide the initial framework for the independent role of MPC2 in homeostasis and diseases related to dysregulated pyruvate metabolism.

## Introduction

Nearly four decades after the demonstration of the protein-mediated transport of pyruvate across the inner mitochondrial membrane (IMM)^1^, two concurrent studies identified the oligomeric complex formed by MPC1 and MPC2 as necessary and sufficient for this task^2,3^; MPC1 and MPC2 were proposed to function together via the formation of an oligomeric structure of approximately 150 kDa^2^. These original findings inspired many subsequent investigations that have further characterized MPC-dependent pyruvate transport in the cellular context^4–6^, with multiple groups showing that either the loss of MPC1 or MPC2 in mitochondria is sufficient to confer similar loss of function phenotypes^6–11^. However, to date, the isolation and reconstitution of MPC1:MPC2 into proteoliposomes is still considered necessary to perform the ultimate proof-of-concept experiment to measure pyruvate transport^9,12,13^.

In this context, we report the first successful, large-scale, recombinant production and functional reconstitution of this family of solute carriers in an artificial lipid bilayer. We provide an unprecedented *in vitro* demonstration that human MPC2 functions independently of MPC1 to induce pyruvate transport, possibly as high-order oligomers. Transport activity is independently demonstrated via both the canonical import of radiolabeled substrate and a novel enzymatic assay that quantifies the decrease of extravesicular pyruvate. In cells, the ectopic expression of human MPC2 improved oxygen consumption and stimulated growth under nutrient-depleted conditions compared to yeast cells lacking endogenous MPC. Most importantly, these observations are consistent with those of early and recent publications, suggesting that mitochondrial pyruvate transport by MPC is a rapid and specific process that depends on co-proton import and redox balance and is sensitive to inhibition by a small molecule^14–17^. Our findings open a discussion concerning pyruvate import regulation by at least two different molecular entities in human mitochondria: heterotypic MPC1:MPC2 and homotypic MPC2:MPC2. Our work also has immediate implications for the development of small-molecule-oriented therapeutics that specifically target MPC2 in pyruvate-related diseases such as cancer, Alzheimer’s disease, and diabetes^9,13,18–21^.

## Results

### Purification of recombinant MPC

**Figure 1:**
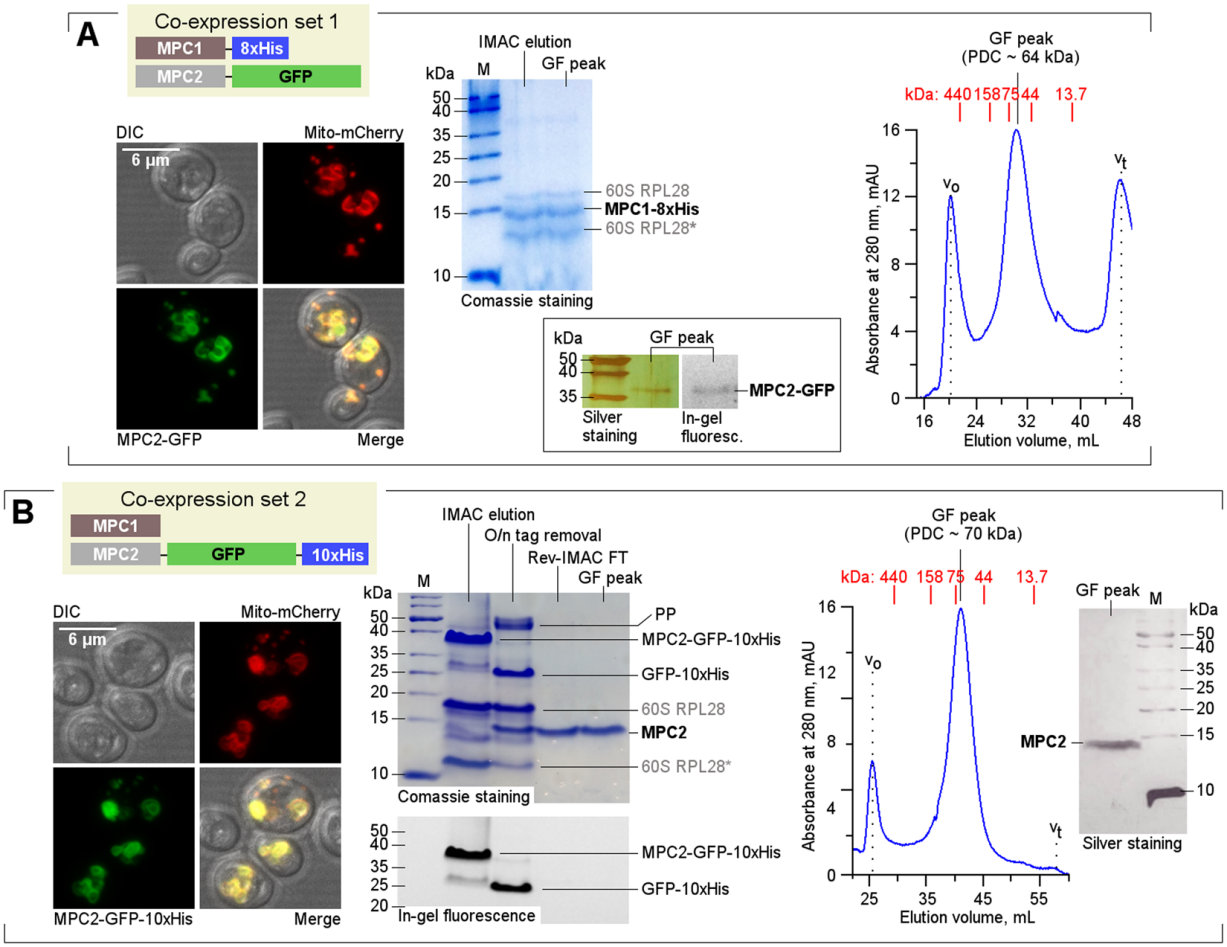
Recombinant expression and purification of human MPC. (**A**) A schematic of the initial human MPC protein constructs expressed in yeast (co-expression set 1). Left panel: Proper localization to the mitochondria was confirmed via confocal microscopy. DIC: Differential interference contrast. Middle panel: Electrophoretic analysis of MPC1-8xHis samples purified using Co-IMAC and GF. *N-terminally truncated portion of 60S RPL28. The inset displays the silver staining and in-gel fluorescence detection of trace amounts of MPC2-GFP, which co-purified with MPC1. Right panel: The corresponding GF trace showed a predominant protein-detergent monodisperse peak at approximately 64 kDa. (**B**) Diagram of the alternative MPC constructs (co-expression set 2) and corresponding confocal microscopy (left panel). Middle panel: Electrophoretic (Tricine-SDS-PAGE) analysis of representative chromatography steps. PP: PreScission Protease. Right panel: GF peak and silver staining analysis revealed that pure monodisperse MPC2 (associated with DDM) was obtained at an equivalent molecular weight of 70 kDa. In all GF profiles above, v_o_ indicates void volume, and v_t_ indicates the total liquid volume of the GF column. The corresponding elution volumes for calibration standards are shown in red. Full-length gels from which silver-stained or fluorescent lanes were cropped are included in Supp. Figure 1.

Human MPC1 and MPC2 proteins were co-expressed from codon-optimized genes in a heterologous yeast system (*Saccharomyces cerevisiae* JRY472) that was modified to lack endogenous MPC (Δ*mpc1/2/3*). This mutant strain is hereafter referred to as 3Δ^2^. A bi-directional expression plasmid was initially engineered to contain MPC1 fused to an 8xHis tag and MPC2 linked to a cleavable monomeric GFP (Fig. 1A). This system enabled the detection of inducible expression levels and cellular compartmentalization as well as subsequent purification via cobalt-based immobilized metal affinity chromatography (Co-IMAC) of the target complex. Controlled expression (Supp. Figure 1A) and proper mitochondrial localization of MPC were confirmed (Fig. 1A, left panel and Supp. Figure 1B). n-Dodecyl-β-D-maltoside (DDM, at 1%) was identified as a suitable detergent for the extraction of proteins from membrane pellets. The target proteins were purified via Co-IMAC, followed by gel-filtration chromatography (GF), both of which were carried out in the presence of 0.03% DDM (Fig. 1A, center panel). A sharp monodisperse peak was obtained after GF, which corresponded to a molecular weight of 64 kDa for the protein-detergent complex (Fig. 1A, right panel). Surprisingly, the peak sample derived from human MPC1 was strongly associated with the yeast 60 S ribosomal protein L28 (both bands were identified using mass spectrometry, Supplementary Table 1). In addition, traces of co-purified, GFP-fused MPC2 were confirmed via silver staining and in-gel fluorescence (Fig. 1A, inset).

Intrigued by the trace amounts of human MPC2 isolated during the complete purification of MPC1, as well as the lower-than-expected molecular weight, we generated an alternative plasmid construct. In this new construct, the poly-histidine tag from MPC1 was transferred to the C-terminus of the MPC2-GFP fusion (Fig. 1B), similar to a previously successful approach employed for other eukaryotic membrane proteins expressed in yeast^22^. Upon confirmation of proper mitochondrial localization of the new construct (Fig. 1B, left panel), the recombinant proteins were successfully extracted with 1% C12E8 (octaethylene glycol monododecyl ether) and purified in the presence of 0.03% DDM via Co-IMAC, followed by GFP-10xHis removal and GF (Fig. 1B, center panel). Again, a single monodisperse protein-detergent peak was obtained, which corresponded to a molecular weight of 70 kDa (Fig. 1B, right panel). This sample was shown to exclusively contain MPC2, which was then purified to homogeneity (Fig. 1B, silver-stained gel; Supplementary Table 2).

### Functional reconstitution of MPC in lipid vesicles

**Figure 2:**
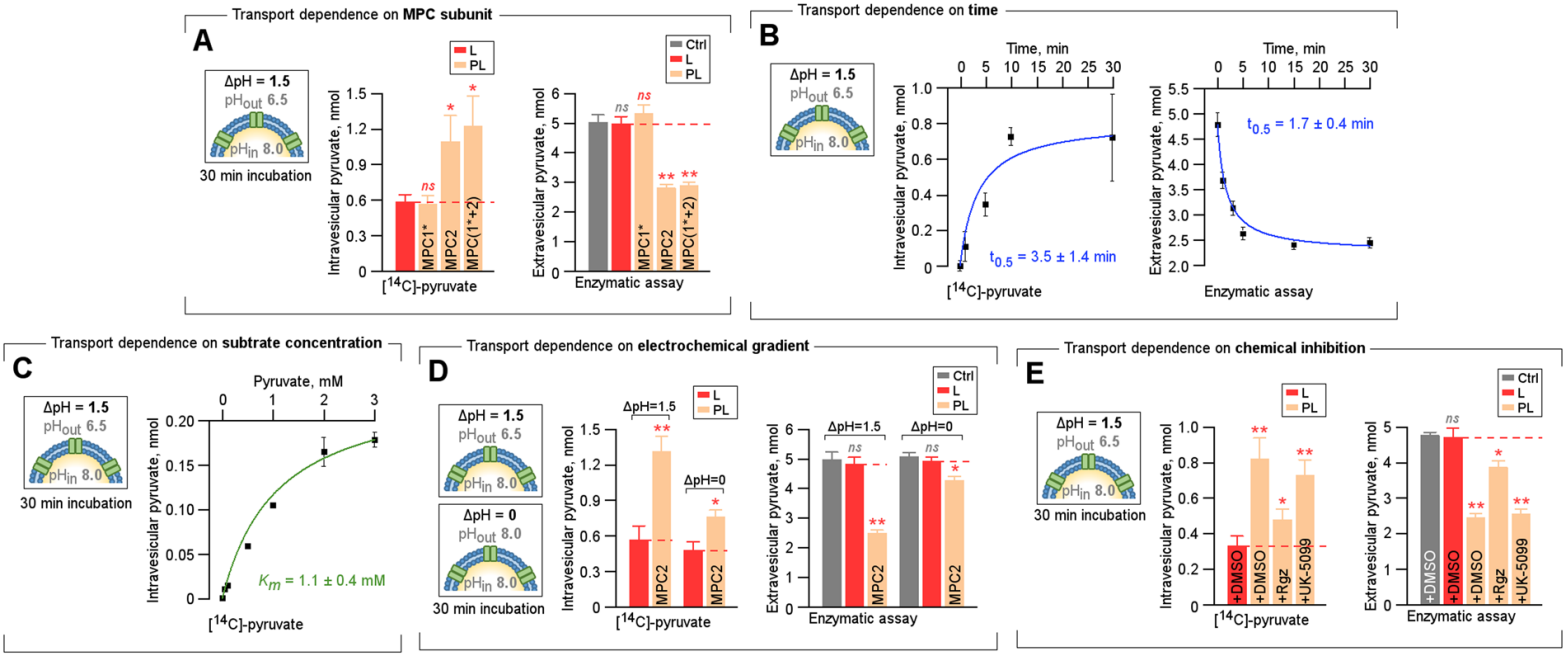
*In vitro* MPC activity and its dependence on time, electrochemical gradient and chemical inhibition. (**A**) Quantification of intravesicular and extravesicular pyruvate, as detected based on ^14^C radiolabeled assay (left panel) and enzymatic assay (right panel), respectively, in liposomes (L) and proteoliposomes (PL) reconstituted with MPC1 and MPC2 and a control condition free of lipid vesicles (Ctrl (-L/PL)). The inset indicates the pH gradient across the outer and inner vesicle environments. An asterisk (*) indicates that MPC1 co-purified with yeast RPL28. (**B**) Quantification of intravesicular and extravesicular pyruvate, as detected based on ^14^C radiolabeled assay (left panel) and enzymatic assay (right panel), respectively in the MPC2-proteoliposome as a function of different incubation times with a ΔpH of 1.5 units. The inset indicates the pH gradient across the outer and inner vesicle environments. Half-maximum times were obtained by the fitting of hyperbolic saturation curves (solid blue lines, R^2^ = 0.99 in both graphs). (**C**) Quantification of intravesicular pyruvate as detected based on ^14^C radiolabeled assay in the MPC2-proteoliposome as a function of different pyruvate concentrations (0.125 mM - 3 mM) with a ΔpH of 1.5 units. The inset indicates the pH gradient across the outer and inner vesicle environments. The Km was obtained by fitting a hyperbolic saturation curve (solid green line, R^2^ = 0.97). (**D**) Quantification of intravesicular and extravesicular pyruvate, as detected based on ^14^C radiolabeled assay (left panel) and enzymatic assay (right panel), respectively in liposomes (L) and MPC2-proteoliposome (PL) after 30 min of incubation for ΔpH = 1.5 and ΔpH = 0. The inset indicates the pH gradient across the outer and inner vesicle environments. (**E**) Quantification of intravesicular and extravesicular pyruvate, as detected based on ^14^C radiolabeled assay (left panel) and enzymatic assay (right panel), respectively in liposomes (L) and MPC2-proteoliposome (PL) in the presence of DMSO (vehicle control) and the well-established MPC inhibitors UK-5099 and rosiglitazone (Rgz). The inset indicates the pH gradient across the outer and inner vesicle environments. For all experiments, results are reported as mean ± standard deviations from triplicates to sextuplicates. Statistical significances were assessed by Welch’s unpaired *t* test, where *ns* = non-significant, *p < 0.05 and **p < 0.001.

To evaluate the pyruvate transport activity of recombinant purified MPC *in vitro*, we reconstituted both purified protein products, individually and in combination, into asolectin liposomes (Supp. Figure 2A,B). The choice for asolectin was based on previous literature^23^. Two independent approaches were adopted to demonstrate the protein-mediated substrate transport: (i) the canonical quantification of the increase in intravesicular ^14^C-labelled pyruvate^2,3,6,15^ and (ii) the quantification of the decrease in extravesicular pyruvate, as indicated by the enzymatic activity of lactate dehydrogenase A (LDHA) (Supp. Figure 3C). A pH gradient-dependent assay (ΔpH = 1.5) was derived in accordance with the chemical conditions recently established for mouse MPC^14^. Notably, successful pyruvate transport activity was not observed for proteoliposomes reconstituted with MPC1 co-purified with RPL28 and traces of MPC2 (termed MPC1*, Fig. 2A and Supp. Figure 3A). By contrast, compared to protein-free liposomes, which were used as a control, proteoliposomes containing MPC2 alone showed significant pyruvate transport activity (Fig. 2A and Supp. Figure 3A). Similarly, vesicles co-reconstituted with comparable levels of MPC1* and MPC2 (Supp. Figure 3D) also induced the transport of pyruvate; however, the corresponding transport levels were similar to those obtained with MPC2 alone (Fig. 2A and Supp. Figure 3A).

Most importantly, the self-sufficient role of human MPC2 in pyruvate transport was further supported when this protein was overexpressed and purified from a plasmid construct lacking the MPC1 coding sequence (Supp. Figure 1C and Supp. Figure 3A). As such, when reconstituted into proteoliposomes, this new construct (termed MPC2**) induced pyruvate transport across the bilayer at levels statistically similar to those mentioned above (Supp. Figure 3A).

### *In vitro* pyruvate transport is dependent on time and electrochemical gradient

Within a pH gradient of 1.5 units, MPC2 proteoliposomes induced rapid pyruvate transport. Pyruvate intake, which exhibited saturation kinetics, was proficient during the first 5 to 10 min of incubation, presenting half-maximum efficiency between 2 to 4 min (Fig. 2B and Supp. Figure 3B). The substrate concentration-dependence of uptake was also studied using radiolabeled substrate. The *K*_*m*_ value for pyruvate of MPC2 in artificial vesicles is 1.1 ± 0.4 mM (Fig. 2C). Similarly, the necessity of MPC2 activity on a proton electrochemical gradient was further confirmed; when the pH gradient was collapsed (ΔpH = 0), a decrease in pyruvate transport activity of about 70 to 80% was observed (Fig. 2D and Supp. Figure 3A). The residual transport activity at ΔpH = 0 likely reflects the chemical potential of pyruvate moving down its own concentration gradient. To quantify the decrease in extravesicular pyruvate as a function of pH, proper enzymatic activity calibration was carried out (Supp. Figure 2C).

### *In vitro* pyruvate transport is sensitive to chemical inhibition, redox modification and substrate type

The previous landmark demonstration of the carrier-dependent transport of pyruvate across the IMM involved the identification of monocarboxylate mimetic cinnamates as effective and specific MPC inhibitors^1^. In the present study, compared to the vehicle control (DMSO), UK-5099, a potent cyanocinnamate (*K*_*i*_ ≈ 10 nM in mitochondrial extracts^24^), did not exert any significant inhibitory effect on *in vitro* MPC2-dependent pyruvate transport when used at a final concentration of 50 µM, as shown in Fig. 2E and Supp. Figure 3D.

On the other hand, when compared to the vehicle control, rosiglitazone (Rgz), at 50 µM, significantly decreased the MPC2-dependent internalization of pyruvate, by over 60%, as shown in Fig. 2E and Supp. Figure 3A. The finding that a thiazolidinedione (TZD) derivative, such as rosiglitazone, can directly and specifically decrease MPC2 activity has been substantiated by previous studies^17,19^; thus, further confirming the protein-mediated component of *in vitro* pyruvate transport.

The inhibitory mechanism of cinnamates was originally shown to rely on a reversible Michael addition to a cysteine thiol group in MPC; loss of conjugation was achieved by the addition of reducing agents such as β-mercaptoethanol^15,25^. Here, although the complete elimination of TCEP (TRIS(2-carboxyethyl)phosphine) from the early steps of membrane preparation up to proteoliposome reconstitution still permitted the standard purification of MPC2 (Supp. Figure 4A), complete functional inactivation was observed (Supp. Figure 3E). Re-incubating MPC2 with TCEP at the stage of proteoliposome reconstitution resulted in the recovery of approximately three quarters of the original activity (Supp. Figure 3E).

To further explore the importance of redox modification on human MPC2 function, we generated a point mutant by replacing the single cysteine residue at position 54 with serine (Supp. Figure 4B). Although the overall activity of the isosteric mutant (termed MPC2.C54S) was compromised, it was distinctly insensitive to TCEP (Supp. Figure 3F). Together, however, these findings suggest that competent pyruvate transport also relies on a sulfhydryl-sensitive site on MPC2.

Finally, among all of the many monocarboxylates metabolized in the cell, lactate and pyruvate are directly linked and structurally similar. However, MPC2 appears to be selective for pyruvate over lactate, as no detectable transport of the latter was observed over the range of concentrations tested and under the chemical conditions shown to be successful for pyruvate (Supp. Figure 3G).

### Ectopic expression of human MPC2 stimulated growth and induced glucose-dependent respiration in yeast

**Figure 3:**
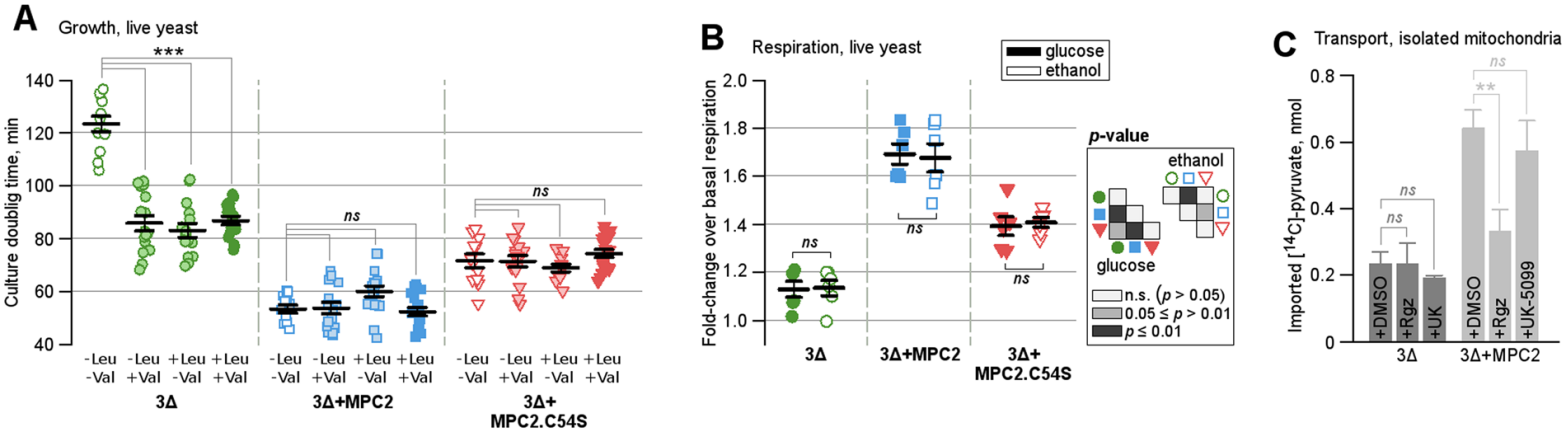
Autonomous transport function of MPC2 *in vivo*. (**A**) Differential growth rates of 3Δ cells or JRY472 bearing an empty plasmid (green circles) or expressing either wild-type MPC2 (blues squares) or MPC2.C54S (red triangles) in the absence and presence of external leucine and valine. Lines indicate the mean ± standard error for three independent experiments. (**B**) Differential respiration rates of 3Δ cells or JRY472 (green circles) expressing either wild-type MPC2 (blues squares) or MPC2.C54S when exposed to glucose (filled geometric shapes) and ethanol (empty geometric shapes). Lines indicate the mean ± standard error for two independent experiments. (**C**) Quantification of imported pyruvate, as detected based on ^14^C radiolabeled assay in isolated mitochondria from 3Δ yeast cells and 3Δ + MPC2 yeast cells in the presence of DMSO (control) and the inhibitors rosiglitazone (Rgz) and UK-5099. Results are reported as mean ± standard deviations from triplicates. In all cases, statistical significances were assessed by Welch’s unpaired *t* test, where *ns* = non-significant, *p < 0.05, **p < 0.001 and ***p < 0.001.

The yeast strain JRY472 (3Δ), used in the present study to produce recombinant human MPC, carries a mutation in the *leu2* gene and is therefore dependent on external leucine to grow. In order to study the metabolic dependences on leucine and valine, as a function of MPC2 activity, cells were co-transformed with the plasmid pYES2 (LEU2). When cultured on synthetic dropout medium lacking both leucine and valine (−Leu −Val), this mutant strain grew with a doubling time of 123 ± 3 min (Fig. 3A, empty green circles). The sole ectopic expression of human MPC2 in this strain, however, decreased the doubling time by 57% (down to 53 ± 2 min; Fig. 3A, empty blue squares). Accordingly, expression of the MPC2 isosteric mutant (C54S), with expected intermediary transport activity compared with wild-type MPC2, showed a 42% decrease in the doubling time of the same strain (71 ± 3 min; empty red triangles in Fig. 3A). Further supplementation of with only leucine (+Leu −Val) or only valine (−Leu +Val), or both amino acids (+Leu +Val), did not alter the doubling time of cells expressing MPC2 or MPC2.C54S (Fig. 3A; light colored shapes for individual nutrients and filled shapes for both nutrients at the same time). However, the individual or combined addition of such amino acids significantly improved the doubling rates of 3Δ cells (bearing empty pBEVY plasmid) to about 85 minutes, or about 31% faster rates. Therefore, the improved growth of 3Δ cells expressing human MPC2 (as well as the isosteric mutant) reflect the mitochondrial use of glycolytic pyruvate for biosynthetic purposes, particularly for the production of branched chain amino acids.

The ability of human MPC2 to stimulate pyruvate-dependent respiration in yeast cells was also evaluated by quantifying oxygen consumption in response to the addition of 0.08% glucose to basal media. Whereas the mutant strain 3Δ responded to glucose addition with a slight, 10% increase in oxygen consumption over basal respiration (1.1 ± 0.1-fold; filled green circles in Fig. 3B), the same cells expressing human MPC2 responded to glucose addition with a 70% increase in oxygen consumption over basal respiration (1.7 ± 0.1-fold; filled blue squares in Fig. 3B). Again, as expected, the ectopic expression of MPC2.C54S led to a reduced increase in oxygen consumption over basal levels (1.4 ± 0.1-fold) in response to glucose addition (filled red triangles in Fig. 3B). To provide further evidence that all the added glucose was being oxidized via respiration, ethanol addition – which blocks residual fermentation – produced nearly the same response under all three test conditions (Fig. 3B, open shapes). Actual respiration rates are depicted in Supp. Figure 5.

Due to issues related to yeast cell wall permeability of both rosiglitazone and UK-5099, the inhibition sensitivity of MPC2 transport in a native environment was most properly addressed when mitochondria were extracted. First and most importantly, by comparing the incorporation of ^14^C-pyruvate into mitochondria isolated from the both 3Δ and 3Δ + MPC2 yeast cells, we confirm that this subunit can play an autonomous role in promoting the active transport of this substrate (Fig. 3C). In addition, the same MPC2 response profile (or lack thereof) to rosiglitazone and UK-5099 as in the artificial vesicle system is herein established (Fig. 3C).

### Oligomeric nature and secondary structure composition of human MPC2

**Figure 4:**
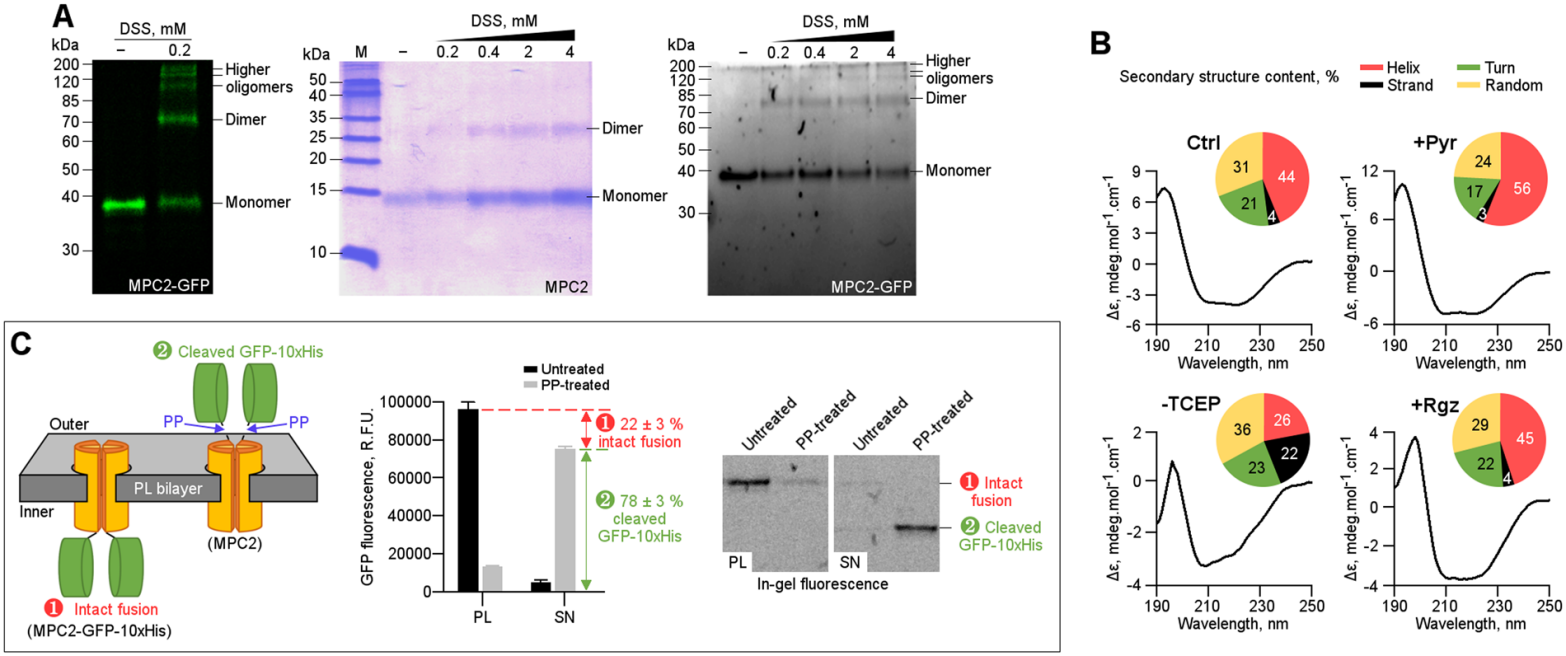
Oligomeric state, secondary structure composition and orientation of MPC2. (**A**) Chemical cross-linking suggested that MPC2 assembled into higher oligomers in a lipid environment. Left panel: Cross-linking of human MPC2 from isolated yeast mitochondrial extracts by 0.2 mM DSS. Middle panel: Cross-linking, using increasing DSS concentrations (0.2–4 mM), of purified MPC2-detergent (DDM) complex. Right panel: Cross-linking, using increasing DSS concentrations (0.2–4 mM), of purified MPC2 reconstituted in asolectin-derived lipid vesicles. (**B**) Secondary structure analysis of MPC2 in presence and absence of ligands, as probed by Circular Dichroism. Far-UV CD spectra, as well as calculated percentage of secondary structure content in the form of pie-chart, for MPC2 alone (Ctrl; top left), in the presence of 25 µM pyruvate (+Pyr; top right), in the absence of TCEP (−TCEP; bottom left), and in the presence of 50 µM rosiglitazone (+Rgz; bottom right). (**C**) Right panel: A schematic representation indicating the possible MPC2-GFP-10xHis orientations in the artificial lipid bilayer of the proteoliposomes, as well as the two possible outcomes upon treatment with PreScission protease (PP): ➊ particles with the C-terminal fusion facing the interior of vesicles are protected from digestion by PP and ➋ cleavage of GFP-10xHis tag when the C-terminal fusion is oriented towards the outside of the lipid membrane. Middle panel: The corresponding samples were quantified in a plate reader for GFP fluorescence, and the relative populations are indicated as percentages. Left panel: Qualitative electrophoretic analysis of the proteoliposomes (PL) and supernatant (SN) samples, before and after treatment with PP.

The lower-than-expected molecular weight of the purified protein-detergent complex that was obtained above (Fig. 1B,C), led us to further investigate the oligomeric assembly of the functional MPC2. First, by isolating the mitochondrial extracts^26^, followed by short-range chemical cross-linking using disuccinimidyl suberate (DSS, 11.4 Å linker), we observed the formation of MPC2 oligomers in its native lipid environment, starting from dimers up to higher-order molecular species, according to electrophoretic separation (Fig. 4A, left panel). When such experiment was performed with purified protein, only the presence of dimeric species was resolved, probably due to the well-known association between the protein and the detergent (Fig. 4A, middle panel)^27^. However, the subsequent reconstitution of purified MPC2 into proteoliposomes displayed the similar high-order, multi-species pattern that was observed in the mitochondrial extracts (Fig. 4A, right panel). This suggests that recombinant MPC2 in artificial lipid bilayer, which was proven functional for pyruvate transport, shares oligomeric similarities to the native mitochondrial membrane environment.

In order to investigate the secondary structure composition of human MPC2, and to gauge the conformational changes due to binding of substrate and inhibitor, we performed synchrotron radiation circular dichroism (SRCD) analysis. SRCD indicated that this protein, in the absence of any ligands, is predominantly helical in nature (Fig. 4B, top left panel). The binding of pyruvate leads to improved structural stability, since the helical content is increased from 44% in the ligand-free control sample up to 56% when the substrate is present (Fig. 4B, top right panel); this might due to the fact that pyruvate needs to be transported across the channel of MPC2, by means of opening up or rearranging the structure. Conversely, the absence of TCEP lead to an overall increased disorder (Fig. 4B, bottom left panel), which is in agreement with the lack of *in vitro* transport activity under a similar condition (Fig. 3B). Notably, the addition of rosiglitazone had minor impact on the secondary structure composition of MPC2 (45% versus 44% helices, respectively; Fig. 4B, bottom right panel), which could probably be associated with weak binding and thereby explaining its limited inhibitory effects on *in vitro* transport (Fig. 2D).

Lastly, targeted digestion^28,29^ of intact MPC2-GFP-10xHis proteoliposomes, followed by fluorescence detection of cleaved GFP-10xHis, revealed that 22 ± 3% of the fused particles were protected from proteolytic processing (Fig. 4C). This indicates that the human MPC2 homotypic complex preferably oriented with the C-terminal end exposed to the outer surface of the artificial bilayer (78 ± 3%); curiously, the dominant orientation is in consensus with the membrane topology proposed for the yeast homolog at the IMM^6^. The current lack of a three-dimensional molecular structure of MPC2 limited our ability to provide a detailed, autonomous mechanism for pyruvate transport. Therefore, experiments are currently in progress to investigate the crystal structure of MPC2.

## Discussion

### Human MPC2 as a potential autonomous transporter

A rich body of evidence has established MPC1 and MPC2 as mandatory components for the mitochondrial uptake of pyruvate in both intact cells and mitochondrial isolates^2,3,5,6,9,10,19^. Co-immunoprecipitation from purified mitochondria, followed by electrophoretic separation, suggested that MPC1 and MPC2 form an oligomeric complex of approximately 150 kDa^2^. Here, we developed an experimental setup aimed at the co-expression and purification of recombinant human MPC as a heterocomplex. To this end, we used a bi-directional expression plasmid with inducible promoters that respond to galactose, such that both isoforms could be expressed simultaneously at high and similar levels. Moreover, we used a mutant yeast strain lacking the endogenous *mpc1*, *mpc2* and *mpc3* genes^2^ to avoid cross-contamination with yeast MPC.

Regardless, only the human MPC2 subunit was purified to homogeneity and shown to function in both *in vitro* and *in vivo* pyruvate transport. This finding was further confirmed when MPC2 was expressed without MPC1 and shown to be similarly functional. MPC1 co-purified with the yeast 60S RPL28 protein and only traces of MPC2. In contrast to MPC2, MPC1 associated with 60S RPL28 was not active. While these results indeed confirm the existence of a MPC1:MPC2 interaction, they suggest that yeast may lack some critical and yet unidentified protein component for stable complex formation of human MPC1:MPC2, as previously proposed^12^. The existence of novel and direct interacting partners for MPC has been experimentally postulated^30,31^; moreover, a host non-membrane protein contaminant (60S RPL28) may act as a chaperone for human MPC1, potentially preventing detectable *in vitro* transport activity. Overall, our findings do not exclude the possibility that, in cells, MPC1 is a co-transporter working with MPC2; however, our data demonstrate that MPC2 can function as an autonomous pyruvate transporter and may work by itself in some specific conditions.

Collectively, the functional observations described above are consistent with previous and recent reports from many laboratories, suggesting that mitochondrial pyruvate transport by MPC is a rapid and specific process that depends on co-proton import and redox balance^14–16^. Our studies on the dependence of time for the transport activity of MPC2 in artificial vesicles (Fig. 2B) agree with those performed early on in mitochondria extracts containing MPC1 and MPC2^15^; half-maximum transport for MPC is achieved around 2 minutes. However, the *K*_*m*_ of MPC2 for pyruvate in vesicles is about 7 times higher than previously reported (1.1 mM in this study versus 0.15 mM quantified by Halestrap, 1975^15^). This could suggest that the MPC2 homo-oligomer is a relatively less efficient transporter than the proposed MPC1:MPC2 heterocomplex.

Here, the finding that the MPC2 autonomous activity can be inhibited by a TZD derivative, albeit partially, is also substantiated by previous literature^17,19^. Of note, these functional parameters, which were proven essential for the demonstration of the protein-dependent pyruvate transport, are further reinforced by the circular dichroism studies using synchrotron radiation. A challenging point, however, is that MPC2 alone is insensitive to UK-5099. Although MPC1, but not MPC2, was recently confirmed to be the true target of cinnamates^2,17^, the description of such a UK5099-insensitive MPC transport in cells remains elusive.

The dynamic stoichiometry of MPC subunits was recently shown to shift between hetero- and homo-oligomers in cells due to variations in both metabolic and tissue-related conditions^6,9,32^. Here, the study of the oligomeric composition of recombinant MPC2 by chemical cross-linking in native (mitochondrial extracts) and native-like (asolectin vesicles) environments, further support the formation of large homotypic assemblies as the functional components, ranging hundreds of kilodaltons, as previously proposed for the heterotypic MPC1:MPC2 complex^2^. The true size of the MPC2 oligomers, however, are yet to be determined.

Given all the potential scenarios described above, a major question arises: which physiological conditions promote MPC2-mediated pyruvate transport? Recently, MPC2 was shown to be strictly regulated by acetylation, which can critically determine the active or inactive states of this protein^11,33–35^; indeed, acetylation has been directly linked to decreased cellular oxygen consumption rates and deficient pyruvate transport activity^33^. Lethality in embryonic mice was caused by either the deletion of MPC2^7^ or the knockout of MPC1, which in turn also lead to the instability of MPC2 at a post-translational level^8^. We showed that human MPC2 alone can increase oxygen consumption in yeast cells driven by glucose addition and can rescue cell growth when valine is absent, suggesting that, in cells, human MPC2 facilitates mitochondrial pyruvate intake. This finding was further confirmed using the isosteric mutant. Curiously, MPC2 significantly increased oxygen consumption in the 3Δ mutant strain by 60%; yet, compared with the control, MPC2 stimulated cell growth rates (measured as doubling time) by 16% in this strain. These results suggest that MPC2 may play a particular role in the distinct energetic and biosynthetic fates of pyruvate, which should be further investigated, perhaps in association to regulation by post-translational modifications.

Our research interest on the metabolic changes undergone by breast cancer led us to survey the MPC2:MPC1 balance in healthy and disease. In particular, we analyzed the MPC2/MPC1 expression ratio in normal and cancer tissues from the Breast Invasive Carcinoma (BRCA) dataset publicly available at The Cancer Genome Atlas project. We noticed that, while normal tissues have a MPC2/MPC1 median ratio near 1, meaning both genes being equally expressed at RNA level, in tumor tissues the ratio is significantly higher, demonstrating an increased unbalanced expression of these genes, with MPC2 ever more abundant (Supp. Figure 6A, shown in log-2 scale). According to a Metacore analysis (Clarivate Analytics) a positive correlation with increase MPC2/MPC1 is observed for 56 out of 80 genes belonging to the oxidative phosphorylation pathway (Supp. Figure 6B). While several of glycolytic genes also positively correlated with higher MPC2/MPC1, there was a consistent negative correlation between MPC2/MPC1 ratio and the expression of five lactate dehydrogenase and gluconeogenesis genes (Supp. Figure 6C). Altogether, a shifted MPC2/MPC1 ratio in breast tumors may have an impact on respiratory metabolism which could lead to increase in the mitochondrial pyruvate oxidative metabolism. This scenario points to the hypothesis that MPC2 may work autonomously for the intake of pyruvate in some specific physiological conditions.

This scenario points to the hypothesis that higher MPC2/MPC1 expression ratio in breast tumors may impact on a more respiratory metabolism with a potential increase in the destination of pyruvate to the mitochondrial oxidative metabolism and suggest that MPC2 may work autonomously for the intake of pyruvate in some specific scenarios. Further research is necessary to identify the biological circumstances under which MPC2 function by itself and in association with MPC1.

In conclusion, although the heterotypic MPC component was initially proposed based on co-immunoprecipitation and yeast-based functional complementation assays^2^, evidences still lack regarding the direct interaction between MPC1:MPC2 at the level of the isolated proteins. In this context, our work provides the first demonstration of the heterologous expression and purification of human MPC1 and MPC2. MPC2, but not MPC1, was purified to homogeneity and found to be active and sufficient for pyruvate transport in artificial liposomes and in cells.

### A transport assay alternative to radiolabeled substrates

The compelling demonstration of the active transport mechanism of pyruvate across the inner mitochondrial membrane, as well as the subsequent identification of proteins responsible for such, were done by using ^14^C-labelled radioactive pyruvate^1–3,6^. This experimental approach effectively confirms substrate entrance into mitochondrial extracts^1–3,6^ and allows for a direct quantification of the parameters involved for transport. In the present work, we introduce a simple, automatable and non-radiometric assay sufficiently sensitive to measure substrate transport into lipid vesicles. As opposed to a more canonical assay which quantifies the intravesicular content of radiolabeled substrate, we propose an alternative novel assay which relies on the observed decrease in the content of extraliposomal pyruvate, as quantified by an enzymatic reaction; in this case, LDHA.

The validity of the enzymatic assay reported here is confirmed when the functional and kinetic parameters of mitochondrial pyruvate transport – collectively reported over the decades – are reproduced using purified MPC and compared side-by-side with radiolabeled substrate. In addition to allowing for a direct and reliable quantification of the key parameters involved in transport, it could be multiplexed and streamlined, therefore adapted to a high-throughput screening approach for modulators of MPC2 using small-molecule libraries.

Therefore, besides the novel functional insights that our paper brings to the field of pyruvate transport, we expect that the successful application of an alternative enzymatic approach to quantify *in vitro* transport will be of the major interest to the *in vitro* biochemistry of membrane carriers in general; this kind of assay can be a suitable replacement to the prevalent radioactive-based assays, provided that the kinetics parameters of the probing enzyme does not become rate limiting in respect to the kinetics of the carrier.

## Materials and Methods

### Construct design and cloning

pBEVY-GU^36^ (bi-directional expression vector for yeast, galactose inducible, URA3 selection) was a generous gift from Graham D. Pavitt (University of Manchester, UK). We are also grateful to Dr. Pascal Egea (University of California, Los Angeles) for the kind gift of alternative vectors from the pBEVY series. Standard molecular biology techniques were used to modify the original pBEVY-GU plasmid to add (i) the coding sequence for an 8xHis tag (with a four-amino acid spacer, VDGS) to the 3′ end of multiple cloning site 1 and (ii) a PreScission Protease recognition sequence, followed by EGFP at the 3′ end of multiple cloning site 2. Codon-optimized, synthetic genes encoding human MPC1 (NCBI Reference Sequence: NP_057182.1) and MPC2 (NCBI Reference Sequence: NP_001137146.1) were acquired from GenScript, USA. The restriction sites SalI/BamHI and XmaI/EcoRI were used to subclone *MPC1* and *MPC2*, respectively. Standard techniques were also used to transfer the poly-histidine tag from the C-terminus of MPC1 to the C-terminus of EGFP. The additional constructs MPC1-mTFP + MPC2-GFP-10xHis, MPC2 alone (plasmid construct devoid of the coding sequence for MPC1), and MPC2.C54S were generated using standard molecular biology techniques. The plasmids were propagated in TOP10 chemically competent bacteria (Thermo Fisher, USA).

### Preparation of competent cells and transformation into yeast

The expression plasmids were transformed into a triple deletion mutant yeast strain (JRY2242, W303 *mpc1Δmpc2Δmpc3Δ*, *his3 lys2 met15 trp1 ura3*)^2^, kindly provided by Jared Rutter (University of Utah, USA). The procedure for transformation was adapted from a previously published protocol^37^. Briefly, one yeast colony was inoculated into 5 mL of yeast peptone dextrose medium (YPD) and cultured overnight in an orbital shaker at 200 rpm, 30 °C. The following day, 0.8 mL of overnight culture was diluted into 30 mL of YPD in a 250 ml flask and grown as above until reaching an OD_600_ of 0.8. The cells were centrifuged at 4,000 *g* for 10 min at 4 °C, and the pellet was resuspended in 25 mL of sterile water. The centrifugation process was repeated, and the competent cells were initially resuspended in 1 mL and then 0.4 mL of 100 mM lithium acetate. Subsequently, transformation was performed on ice, unless otherwise mentioned. To the 50 μL of competent cells 240 μL of 50% (w/v) PEG 3350 was added for each transformation. A total of 25 μl of 2 mg.mL^−1^ single-stranded carrier DNA was added to each tube and incubated for 5 s. Subsequently, 50 μL of DNA encoding human pyruvate carrier (100–150 ng) diluted in sterile water was added to each tube and incubated for 5 s, followed by heat shock for 45 min at 42 °C. The cells were centrifuged at 8,000 *g* for 1 min at RT, and the pellet was resuspended in 100 μL of sterile water. The cell suspension was plated onto SD-URA plates and incubated for 3 days at 30 °C.

### Protein expression and purification

Typically, for large-scale overexpression of recombinant MPC, colonies from the SD-URA plates with the highest fluorescence levels were selected and used as pre-inoculum, with large-scale cultures (typically 4 L) grown in shake flasks. Two hundred-milliliter cell cultures (in 500 mL flaks) transformed with MPC plasmids were grown overnight in YPD (2% glucose) with shaking at 200 rpm at 30 °C. The following morning, 10 mL of overnight culture was added to 500 mL of YPD (0.2% glucose) in a 2 L flask and cultured as above until the OD_600_ reached 0.8. Then, 2% galactose (from a 20% stock) was added to the culture. After 22 h, the cells were harvested via centrifugation at 4,000 *g* for 10 min. The pellets were suspended in 400 mL of buffer containing 1 × PBS, supplemented with 150 mM NaCl, 10% glycerol, 2 mM β-mercaptoethanol and a protease inhibitor mix. Cell lysis was performed using a benchtop cell disruptor (Constant Systems) at increasing pressures (25, 28, 30, 32, 35 and 38 kpsi). After lysis, the cell suspension was centrifuged at 10,000 *g* for 10 min at 4 °C. The supernatant was retained and further ultra-centrifuged at 150,000 *g* for 2 h to pellet the membranes. Isolated membranes of MPC1-8xHis or MPC2-10xHis were resuspended in similar solubilization buffer (SB) containing 1 × PBS, pH 8.0, and 150 mM NaCl; 0.5 mM TCEP: 1% (w/v) C12E8 was used for MPC2-10xHis, and 1% (w/v) DDM was used for MPC1-8xHis. Membrane-detergent mixtures at a 2:1 ratio were rotated for 1 h at 4 °C. The suspension was cleared via ultra-centrifugation at 100,000 *g* for 45 min, and 10 mM imidazole was added to the solubilized solution. The solubilized samples were incubated for 3 h at 4 °C with TALON beads (Clontech) pre-equilibrated with SB (supplemented with 0.1% (w/v) C12E8 + 0.1% (w/v) DDM for MPC2-GFP-10xHis and with 0.1% (w/v) DDM for MPC1-8xHis). Following incubation, the sample was loaded onto a glass column (Bio-Rad) and washed with 20 column volumes (CVs) of SB containing 0.1% (w/v) DDM and 15 mM imidazole. The column was subsequently washed with another 20 CVs of the same buffer containing 30 mM imidazole. Bound proteins were eluted with SB containing 0.1% (w/v) DDM and 300 mM imidazole. A similar procedure of column washing and elution was followed for both MPC2-10xHis and MPC1-8xHis. The eluted protein from the MPC1-8xHis construct was collected, concentrated to 5 mg.mL^−1^ and immediately loaded onto a Superdex 200 PG 16/30 column (GE Healthcare, Sweden) pre-equilibrated in 20 mM TRIS, pH 8.0, 150 mM NaCl, 0.5 mM TCEP and 0.03% DDM. The protein eluted from MPC2-10xHis construct was dialyzed overnight against 20 mM TRIS, pH 8.0, 300 mM NaCl, 5% glycerol, 0.05 mM TCEP and 0.03% DDM in a 6,000-Da MWCO dialysis bag (Spectrum Laboratories, USA) in the presence of 10:1 (GFP fused pyruvate carrier: protease) GST-tagged PreScission Protease at 4 °C. After dialysis, the digest was passed through TALON (Clontech, USA) and GST (GE Healthcare, USA) beads equilibrated in dialysis buffer. The flow-through containing the membrane protein was collected and concentrated to 5 mg.mL^−1^ and loaded directly onto a Superdex 200 PG 16/30 column previously equilibrated with 20 mM TRIS, pH 8.0, 300 mM NaCl, 5% glycerol, 0.05 mM TCEP and 0.03% DDM. MPC2-10xHis in the absence of TCEP, and additional constructs, such as MPC2 alone (plasmid construct devoid of the coding sequence for MPC1) and the MPC2.C54S mutant, were similarly purified as described above. For all MPC constructs used in the present study, a typical purification preparation starting with inducible expression from four liters of yeast culture, resulted in approximately 1 mg of pure protein sample after full purification. Electrophoretic analysis was performed using Tricine-SDS-PAGE.

### Confocal Microscopy

The Mito-mCherry plasmid for yeast expression used for mitochondrial labeling was kindly provided by Jean-Claude Martinou (Université de Genève, Switzerland). Yeast co-transformed with either of the MPC-expression plasmids or Mito-mCherry were cultured under the same conditions described above to facilitate the expression of GFP-labeled MPC. Subsequently, 10 mL of 22-h induced cells (at OD_600_ of 1) was centrifuged at 250 rpm for 5 min. The pellet was resuspended in 1 mL of YPD. From this, 7 µL of cells were added to a slide and sealed with a cover slip. Samples were examined in the Biological Imaging Facility (LIB) at LNBio, using a Leica TCS SP8 confocal on a Leica DMI 6000 equipped with an HC PL APO 63×/1.40 Oil CS2 objective.

### Quantitative PCR

Total RNA was extracted using the TRIzol reagent (Sigma, US), and 5 µg of total RNA was used for retrotranscription using GoScript™ Reverse Transcription System (Promega). Real-time quantitative PCR for *MPC1* and *MPC2* was performed using the SYBR Green PCR Master Mix (Applied Biosystems). The threshold cycle (CT) values of the target genes were normalized to the actin gene, and relative expression ratios were calculated using the 2-ΔΔCT Ct method^38^. Next, the readings were normalized to the URA3 transcripts to calculate the levels of transformed plasmids. The primer sequences were as follows: (1) Actin: ACT1.qPCR.For: 5′–ATTCTGAGGTTGCTGCTTTGG–3′ and ACT1.qPCR.Rev: 5′–TGTCTTGGTCTACCGACGATAG–3′; (2) URA3: URA3.qPCR.For: 5′–TGGCAGCAACAGGACTAGGATG–3′ and URA3.qPCR.Rev: 5′–CGAACAGAAGGAAGAACGAAGGAAG–3′; (3) Human MPC1: hMPC1.qPCR.For: 5′–TGACCTTTGCTTTGTGTTGC–3′ and hMPC1.qPCR.Rev: 5′–CACCTTGGATCAATTGTGCT–3′; (4) Human MPC2: hMPC2.qPCR.For: 5′–CGATATGGCTAGACCAGCAG–3′ and hMPC2.qPCR.Rev: 5′–ACAGCGAACAAAGACCAATTT–3′.

### Fluorescence size-exclusion chromatography (FSEC)

The appropriate detergent for protein extraction from membrane pellets was determined by FSEC analysis^37^. A total of 250 µL of solubilized membranes containing the overexpressed EGFP-fused pyruvate carrier in DDM (Anatrace, OH, USA) for MPC1 and C12E8 (Anatrace, OH, USA) for MPC2 was loaded onto a Superdex 200 PG 16/30 column. The column was pre-equilibrated with 20 mM TRIS, pH 8.0, 150 mM NaCl, 0.5 mM TCEP and 0.03% DDM for MPC1-8xHis and 20 mM TRIS, pH 8.0, 300 mM NaCl, 5% glycerol, 0.5 mM TCEP and 0.03% DDM for MPC2- and connected to an AKTA start FPLC system at 4 °C. The flow rate was set at 1 mL.min^-1^. Fractions of 200 µL were drawn from the elution into 96-well optical plate. GFP emission was measured at 512 nm on bottom read, with an excitation wavelength of 488 nm, using an EnSpire microplate reader (Perkin Elmer, Massachusetts, USA).

### Calibration of gel filtration columns

Standard proteins from the low and high molecular weight Gel Filtration calibration kits (GE Healthcare, USA) were used. Protein resuspension buffer and running buffer were 20 mM TRIS, pH 8.0, 300 mM NaCl, 5% glycerol, 0.5 mM TCEP and 0.03% DDM.

### Mass spectrometry

The protein bands from gel-filtration-purified samples were extracted from tricine-SDS-PAGE gels. The gels were first stained with Coomassie dye for band identification and excision. Bands were then isolated and destained in methanol (50%)/acetic acid (2.5%) solution, and the samples were reduced, alkylated and digested with trypsin^39^. Peptides (4.5 µL) were separated by a C18 (75 µm x 100 mm, 1.7 μm particle size) nanoUPLC (nanoAcquity, Waters) coupled with a Q-Tof Premier mass spectrometer (Waters) with a nanoelectrospray source at a flow rate of 0.6 µL.min^-1^. The gradient was 2–35% acetonitrile in 0.1% formic acid over 31.6 min for the digested proteins. The nanoelectrospray voltage was set to 3.5 kV, with a cone voltage of 30 V, and the source temperature was 80 °C. The instrument was operated in the ‘top three’ mode, in which one MS spectrum is acquired, followed by MS/MS of the top three most-intense peaks detected. After MS/MS fragmentation, the ion was placed on an exclusion list for 60 s. The spectra were acquired using MassLynx v.4.1 software, and the raw data files were converted to a peak list format (mgf) using Mascot Distiller v.2.4.0.0 software 2011 (Matrix Science Ltd.) and were searched against the Human UniProt-SwissProt database (92,180 sequences; 36,693,332 residues; release date March 2016) and the Yeast UniProt-SwissProt database (550,299 sequences; 196,347,838 residues; release date March 2016), with *Saccharomyces cerevisiae* taxonomy (7,743 sequences) using Mascot engine v.2.3.2 (Matrix Science Ltd.) with carbamidomethylation as a fixed modification, oxidation of methionine as a variable modification, one trypsin missed cleavage and a tolerance of 0.1 Da for both precursor and fragment ions.

### Preparation of liposomes and proteoliposomes

The purified MPC protein samples were reconstituted into liposomes using previously described procedures^40,41^ with minor modifications. Briefly, asolectin from soybean extract (Sigma Aldrich) was dissolved in chloroform and dried into a thin film by blowing gaseous N_2_. This film was then resuspended to a final concentration of 4 mg.mL^−1^ in buffer containing 20 mM TRIS, pH 8.0, 300 mM NaCl, 5% glycerol, 0.5 mM TCEP and 0.03% (w/v) DDM and heated at 70 °C for 1 h with vortexing every 10 min. This suspension was sonicated in a water-bath sonicator at 40 kHz for 1 min to obtain the homogenized lipid-detergent solution. The purified proteins were added to the solution at a lipid-to-protein ratio of 10:1 (w/w), along with 0.05% (w/v) DDM to avoid protein denaturation. The ternary protein-lipid-detergent mixture was freeze-thawed three times for full reconstitution and then sonicated for 1 min under the aforementioned conditions. To remove the detergent and allow proteoliposome formation, Bio-Beads SM-2 resin (Bio-Rad) was added at a ratio of 1:0.2 (w/w) and incubated at 4 °C with exchange after 5, 10 and 40 min, followed by ultra-centrifugation of the suspension at 100,000 *g* for 45 min, and the final proteoliposome pellet was resuspended in 20 mM TRIS, pH 8.0, 300 mM NaCl, 5% glycerol, and 0.5 mM TCEP. Protein-free liposomes (small unilamellar vesicles, or SUVs) were prepared following a similar procedure, except that the protein solution was replaced with the gel filtration buffer. Purified MPC2–10xHis in the absence of TCEP, as well as the purified proteins from the additional constructs, such as MPC2 alone (plasmid construct devoid of the coding sequence for MPC1) and the MPC2.C54S mutant, were reconstituted into liposomes following the above protocol. The size and homogeneity of the liposomes and proteoliposomes were assessed via dynamic light scattering and Nanoparticle Tracking Analysis (NTA, Supp. Figure 2A) as described below. To check for unwanted bursting or swelling of the liposomes and proteoliposomes upon incubation with the pyruvate solution, dynamic light scattering analysis was performed (Supp. Figure 2B).

### Nanoparticle Tracking Analysis

The size, homogeneity and concentration of the liposomes and proteoliposomes were assessed via NTA, as available on the NanoSight instrument (Malvern) Dynamic light scattering measurements were performed on a NanoSight NS300 (Malvern Instruments Ltd., United Kingdom). Fifty microliters of total proteoliposomes was diluted in 1 mL of filtered buffer (20 mM TRIS, pH 8.0, 300 mM NaCl, 5% glycerol, 0.5 mM TCEP); 500 µL of this suspension was injected into the sample chamber using a sterile syringe. All measurements were performed at 18 °C, and the average of quintuple readings was determined.

### *In vitro* pyruvate transport: radiolabeled substrate

Radiolabeled pyruvate transport experiments were performed as described previously^2,3,6,15^ with minor modifications. The transport assay was initiated by the addition of 50 µL liposome/proteoliposomes samples with 50 µL of radiolabeled ^14^C-pyruvate at 0.1 μCi dissolved in transport buffer containing 120 mM KCl, 100 mM MES pH 6.5. Upon incubation at desired time points (1, 5, 10 and 30 minutes), at room temperature, the transport assay was stopped by adding 900 µL of 50 mM cold pyruvate in PBS pH 7.4. The vesicles were centrifuged at 15,000 g for 30 minutes followed by PBS wash two times. To the resulting pellet, 1 ml of scintillation buffer was added, and the imported ^14^C-pyruvate was quantified by scintillation counter. To demonstrate the pH dependence on the transport, 50 µL of the liposome/proteoliposomes were added with radiolabeled pyruvate in buffer containing 120 mM KCl, 100 mM TRIS pH 8.0 and incubated for 30 minutes at room temperature. The reaction was stopped and the measurement of imported ^14^C-pyruvate was carried out as described above. Further, to validate the inhibition of pyruvate transport 50 µL of the liposome/proteoliposomes samples were pre-incubated for 30 minutes with UK5099 and rosiglitazone dissolved in 0.1% DMSO at a final concentration of 50 µM respectively. To these samples radiolabeled pyruvate in transport buffer containing 120 mM KCl, 100 mM MES pH 6.5 was added and incubated for 30 minutes at room temperature. By following the procedure described above the reaction was stopped and the imported ^14^C-pyruvate was quantified by scintillation counter. Background values in each experiment were measured by simultaneous addition of ^14^C pyruvate and its corresponding stop solution.

### *In vitro* pyruvate transport: enzymatic assay

Transport analysis was performed at room temperature and initiated by mixing 50 µL of the liposome/proteoliposome samples with 100 µL of 50 µM sodium pyruvate in a 96-well flat bottom Greiner plate. To induce a pH gradient of 1.5 units between the inner and outer vesicle environments, sodium pyruvate (Sigma, USA) was resuspended at the desired concentration in transport buffer containing 120 mM KCl and 100 mM MES, pH 6.5. Upon incubation for the desired times (1, 3, 5, 15 and 30 min) at room temperature, 50 µL of stop buffer (2 nM LDHA, 0.5 mM NADH, 50 mM TRIS, pH 8.0) was added. The mixture was immediately subjected to quantification of absorbance at 340 nm on an EnSpire plate reader (Perkin Elmer, USA) to track the oxidation of NADH. In addition, the necessity of a collapsed proton electrochemical gradient was measured as described above with minor modifications. Briefly, sodium pyruvate was also dissolved in transport buffers containing 120 mM KCl, 100 mM TRIS, pH 8.0 (for ΔpH = 0). Raw absorption readings were converted to amounts of NADH using an extinction coefficient of 6,220 M^−1^.cm^−1^. Initial velocities for LDHA under all conditions were obtained using linear regression, keeping unrestrained intercepts. When appropriate, analysis of covariance (ANCOVA) was performed to determine whether the difference between two similar slopes was statistically non-significant (indicated as n.s. in the figures and respective legends). Velocities were rounded up to the first significant digit. The obtained enzyme velocities for each transport condition were compared with an LDHA standard curve (Supp. Figure 2C) generated under the same chemical conditions used in the assay but in the absence of liposomes or proteoliposomes, termed Ctrl (-L/PL). Spontaneous oxidation of NADH was shown not to occur during the experimental timeframe (Supp. Figure 2E). The plasmid for bacterial expression of human LDHA was a kind gift from Anne Le (Johns Hopkins Medicine, USA). Experiments to demonstrate the MPC2 pyruvate transport inhibition were performed at room temperature. Fifty microliters of liposome/proteoliposome samples was pre-incubated for 30 min with UK5099 and rosiglitazone dissolved in 0.1% DMSO at a final concentration of 50 µM. As a control, liposome/proteoliposome samples were also similarly incubated with 0.1% DMSO.

### Isolation of yeast mitochondria

Mitochondrial isolation was carried out according to the procedure described^42^ with minor modifications. Briefly, 100 ml of 3Δ yeast with MPC2 was grown in YPD medium for 22 hours at 200 rpm, 30 °C. Cells were recovered by centrifugation for (3,000 *g* 5 minutes) at 4 °C washed once in water and resuspended in 1 ml of ice cold 20 mM HEPES pH 7.4, 0.6 M sorbitol and 1 mM PMSF buffer at 0.5 g cells/ml. Glass beads of approximately two-thirds the final volume was added and the cells were broken by vortex for 15 s. This process is repeated for three times after chilling on ice for every 15 s. The resulting suspension was centrifuged at 600 g for 5 min and the obtained supernatant is carefully transferred to a fresh tube and further centrifuged at 10,000 g for 10 min. The resulting pellet containing mitochondria were resuspended in 1 ml of 20 mM HEPES pH 7.4, 80 mM KCl, 5 mM MgCl_2_ and 250 mM sucrose. The presence of MPC2 in the resulting pellet was further confirmed by tracking GFP emission measured at 512 nm using Enspire (Perkin Elmer, Massachusetts, USA). Similar protocol was followed to isolate the mitochondria of 3Δ yeast cells in the absence of MPC2.

### Quantification of transport using ^14^C pyruvate in isolated mitochondria

Two hundred and fifty micrograms of isolated mitochondria of 3Δ yeast in the absence and presence of MPC2 was added with radiolabeled pyruvate at a similar concentration as described above in transport buffer containing 120 mM KCl, 100 mM MES pH 6.5 and incubated for 30 minutes at room temperature. The uptake was stopped by adding 900 µL of 50 mM cold pyruvate in PBS pH 7.4. The mitochondria were centrifuged at 15,000 *g* for 5 minutes followed by PBS wash two times. To the resulting pellet 1 mL of scintillation buffer was added and the imported ^14^C-pyruvate was quantified by scintillation counter. In order to corroborate the inhibition of pyruvate transport, mitochondria were pre-incubated for 30 minutes with UK5099 and rosiglitazone in a related way as it was carried out for vesicles. The inhibitor bound mitochondria were supplemented with radiolabeled pyruvate in transport buffer containing 120 mM KCl, 100 mM MES pH 6.5 and incubated for 30 minutes at room temperature. The reaction was stopped and the measurement of imported ^14^C-pyruvate was carried out as described above.

### Substrate specificity assay

Lactate was assessed using the L-lactate colorimetric assay kit from Abcam, Inc. (ab65331) according to the manufacturer’s instructions, with minor modifications. Substrate specificity analysis was performed at room temperature and initiated by mixing 50 µL of the liposome/proteoliposome samples with 100 µL of 50 µM sodium lactate (Sigma, USA) in a 96-well flat-bottom Greiner plate. Sodium lactate (Sigma, USA) was suspended to the desired final concentrations of 30, 60 and 120 µM in transport buffer containing 120 mM KCl, 100 mM MES, pH 6.5, which was also used to dissolve sodium pyruvate. Upon incubation for 30 min at room temperature, 50 µL of the lactate enzyme mix provided with the kit was added, and the plate was measured at 450 nm. Data processing and analysis were performed as described above for pyruvate transport. The obtained enzyme velocities for each transport condition were compared with a lactate standard curve obtained in a vesicle-free solution, termed Ctrl (-L/PL). The experiments were repeated four to six times, and the means ± standard deviations obtained were graphically represented.

### Chemical cross-linking of MPC2

Cross-linking of MPC2 in mitochondrial extracts was performed according as previously published^6^, but using DSS instead. For crosslinking of purified protein and protein reconstituted in proteoliposomes, MPC2 was prepared as described above, except that HEPES was used instead of TRIS in the final gel filtration buffer (20 mM HEPES, pH 8.0, 300 mM NaCl, 5% glycerol, 0.05 mM TCEP and 0.03% DDM). Approximately 10 μg of protein was used in each reaction and incubated with DSS at 0.2, 0.4, 2 and 4 mM for 30 min at room temperature. The reactions were stopped using 50 mM TRIS, pH 8.0.

### Growth assays

Yeast cells co-transformed with pBEVY and pYES2 plasmids were grown overnight and inoculated in 5 mL of SD-URA medium supplemented with 0.2% glucose for 22 h at 30 °C, 200 rpm. Cells were recovered by centrifugation for 5 min at 3000 *g*, 4 °C, washed twice and then resuspended in 2.5 mL of SD-URA-LEU-VAL supplemented with 0.2% glucose. OD_600_ was adjusted to 0.034 (approximately 0.23 at 10 mm path-length) for all conditions using an EnSpire Plate Reader (Perkin-Elmer). Then, 10 µL of each cell culture was added to a 96-well plate containing 200 µL of SD-URA (supplemented with 0.2% glucose + 0.2% galactose) in the presence and absence of leucine and valine, or a combination of both. Optical density was measured every 5 min, while cells were left to grow inside the EnSpire Plate Reader, with shaking at 200 rpm, at 30 °C. The pYES2 plasmid was a kind gift from Dr. Celisa Caldana (LNBio).

### Oxygen consumption measurements

Yeast cells were grown overnight and inoculated on 10 mL of SD-URA medium supplemented with 0.2% glucose for 22 h at 30 °C, 200 rpm. Cells were recovered via centrifugation for 5 min, 3.000 *g* at 4 °C and resuspended in 1 mL of 25 mM HEPES-Buffered DMEM, pH 7.1 (Sigma, D5030). OD_600_ was adjusted to 0.034 (approximately 0.23 at 10-mm path-length) for all conditions using an EnSpire Plate Reader (Perkin-Elmer), and cells were centrifuged and resuspended again in the same buffer to remove any residual glucose. Measurements were performed using an Oxytherm (Hansatech, Norfolk, UK). Briefly, 970 µL of buffered DMEM was pre-warmed to 30 °C with shaking at 100 rpm in a reaction chamber. Next, 30 µL of OD-standardized cells was added, and the chamber was equilibrated open for 1 min. The chamber was then closed and incubated for 8 min to measure the basal respiration, followed by another 8 min for glucose (0.08% final concentration to induce mitochondrial oxidation of pyruvate and not fermentation to ethanol^43^), and the final 8 min for ethanol (2% final concentration to inhibit any residual fermentation^44,45^). The raw oxygen concentration in the chamber collected every second was used to calculate multiple linear regressions of 150 s in length and average the oxygen consumption rate using the R software package.

### Circular Dichroism

Synchrotron Radiation Circular Dichroism experiments were performed using a nitrogen-flushed Module X end-station spectrophotometer at B23 Beamline at the Diamond Light Source, Oxfordshire, UK^46^, under proposal SM16289-2.

### Determination of MPC2-GFP-10xHis orientation in lipid bilayer membrane

Triplicate 400 µl aliquotes of proteoliposomes were prepared, with one control sample left untreated. PreScission protease at a ratio of 1:10 (protease: GFP fused pyruvate carrier) was added. All the samples were left gently shaking overnight at 4 °C and ultracentrifuged at 100,000 g for 45 min the following day. The supernatant from the protease un-treated and treated along with the control proteoliposomes were loaded into 96 well plate to measure the GFP emission using Enspire (Perkin Elmer, Massachusetts, USA). Results were normalized to the intensity observed in the presence of PreScisison protease and the percentage digested was calculated and represented graphically. Further, the similar set of samples were resolved on a 15% Tricine gel and imaged by using a CCD camera after exposure to blue light at 460 nm with a 515 nm filter cutoff, for 0.2 s.

### TCGA data analysis

Level 3 TCGA RNA-Seq data (FPKM upper-quartile normalized) for 1093 tumor tissues from the Breast Invasive Carcinoma (BRCA) dataset, as well as 112 normal breast tissue samples, were downloaded from the Genomic Data Commons (National Cancer Institute) on October 21, 2017. Correlations between MPC2/MPC1 ratios were calculated using Pearson coefficient.

## Electronic supplementary material

Supporting Information
