# Supplementary material for: Human mitochondrial pyruvate carrier 2 as an autonomous membrane transporter
Source: Sci Rep. 2018 Feb 22;8:3510. doi: 10.1038/s41598-018-21740-z (PMC5823908; doi:10.1038/s41598-018-21740-z)
Supplement: Supplementary file 1 — Supporting Information [file 41598_2018_21740_MOESM1_ESM.pdf]

SUPPLEMENTARY INFORMATION

Human mitochondrial pyruvate carrier 2 as an autonomous membrane transporter

Raghavendra Sashi Krishna Nagampalli[1,*], José Edwin Neciosup Quesñay[1,*], Douglas Adamoski[1,*], Zeyaul Islam[1], James Birch[2,3], Heitor Gobbi Sebinelli[4], Richard Marcel Bruno Moreira Girard[5], Carolline Fernanda Rodrigues Ascenção[1], Angela Maria Fala[1ε], Bianca Alves Pauletti[1], Sílvio Roberto Consonni[1‡], Juliana Ferreira de Oliveira[1], Amanda Cristina Teixeira Silva[1], Kleber Gomes Franchini[1], Adriana Franco Paes Leme[1], Ariel Mariano Silber[5], Pietro Ciancaglini[4], Isabel Moraes[2,3£], Sandra Martha Gomes Dias[1†] & Andre Luis Berteli Ambrosio[1†]

From the [1]Laboratório Nacional de Biociências, Centro Nacional de Pesquisa em Energia e Materiais, Campinas, SP, 13083-970, Brazil; the [2]Membrane Protein Laboratory, Diamond Light Source, Harwell Science and Innovation Campus, Didcot, Oxfordshire OX11 0DE, England; [3] Research Complex at Harwell, Rutherford Appleton Laboratory, Harwell, Didcot, Oxfordshire OX11 0FA, England; the [4]Departamento de Química, Faculdade de Filosofia, Ciências e Letras de Ribeirão Preto, Universidade de São Paulo, Ribeirão Preto, SP, 14040-901, Brazil; the [5]Laboratory of Biochemistry of Tryps – LaBTryps, Departamento de Parasitologia, Instituto de Ciências Biomédicas, Universidade de São Paulo, São Paulo, SP, 05508-900, Brazil;

*RSKN, JENQ and DA contributed equally to this work.

[ε]current address: Structural Genomics Consortium (SGC), Universidade Estadual de Campinas, Campinas, SP, 13083-886, Brazil.

[‡]current address: Departamento de Bioquímica e Biologia Tecidual, Instituto de Biologia, Universidade Estadual de Campinas, Campinas, SP, 13083-862, Brazil.

[£]current address: National Physical Laboratory, Teddington, Middlesex, TW11 0LW, England.

†To whom correspondence may be addressed: Sandra MG Dias or Andre LB Ambrosio, Laboratório Nacional de Biociências (LNBio), Centro Nacional de Pesquisa em Energia e Materiais (CNPEM), Rua Giuseppe Máximo Scolfaro, 10.000, Pólo II de Alta Tecnologia, Campinas, SP, 13083-970, Brazil. Tel: +55 19 3512 1115/Fax: +55 19 3512 1004; e-mail: sandra.dias@lnbio.cnpem.br or andre.ambrosio@lnbio.cnpem.br

KEYWORDS: membrane protein, mitochondria, transporter, pyruvate carrier, MPC1, MPC2.

Supplementary Table 1. Identification of the purified protein bands by liquid chromatography coupled to tandem mass spectrometry (LC-MS/MS).

Co-expression strategy and the respective bands identified

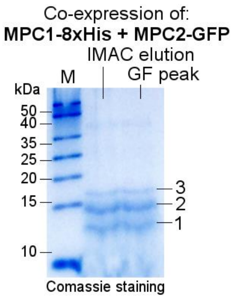

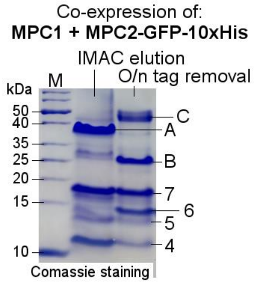

Bands identified by LC-MS/MS

Band	Protein	Database (accession number)	Sequence coverage, %	# of matches	Top-three highest score peptide sequences
1	60S RPL28	Yeast (P02406)	49	42	ETAPVIDTLAAGYGK<br>SASKETAPVIDTLAAGYGK<br>IPNVPVIVK
2	MPC1	Human (Q9Y5U8)	70	43	NWLLFACHATNEVAQLIQGGR<br>DYLMSTHFWGPVANWGLPIAAINDMK<br>SPEIISGR
3	60S RPL28	Yeast (P02406)	53	79	ETAPVIDTLAAGYGK<br>SASKETAPVIDTLAAGYGK<br>INMDKYHPGYFGK
4	60S RPL28	Yeast (P02406)	46	38	ETAPVIDTLAAGYGK<br>GRIPNVPVIVK<br>IPNVPVIVK
5	60S RPL28	Yeast (P02406)	49	51	ETAPVIDTLAAGYGK<br>SASKETAPVIDTLAAGYGK<br>GRIPNVPVIVK
6	MPC2	Human (O95563)	73	107	LSTAQSAVLMATGFIWSR<br>LLDKVELMLPEK<br>TVFFWAPIMK
7	60S RPL28	Yeast (P02406)	53	79	ETAPVIDTLAAGYGK<br>SASKETAPVIDTLAAGYGK<br>INMDKYHPGYFGK

Other relevant bands (not by LC-MS/MS)

Band	Protein	Means of detection
A	MPC2-GFP-10xHis	Inducible expression, Detected by In-gel fluorescence
B	GFP-10xHis	Cleavage product, Detected by In-gel fluorescence
C	PreScission Protease	Artificially added for tag cleavage

Supplementary Table 2. Mass spectrometry identification of yeast proteins co-purified with human MPC2 (as in main text Figure 1B).

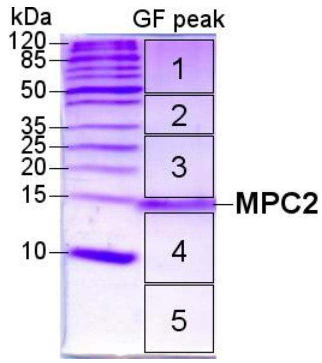

Region	Matches	Expected MW	Uniprot ID	Sequence coverage, %	Number of matches
**1**	Peptide matches not assigned to protein hits				
**2**	Zuotin	49	P32527	3	2
**3**	RANBP1	23	P41920	3	1
**4**	Peptide matches not assigned to protein hits				
**5**	60 RPL28	17	P02406	24	5

Supplementary Figure 1.

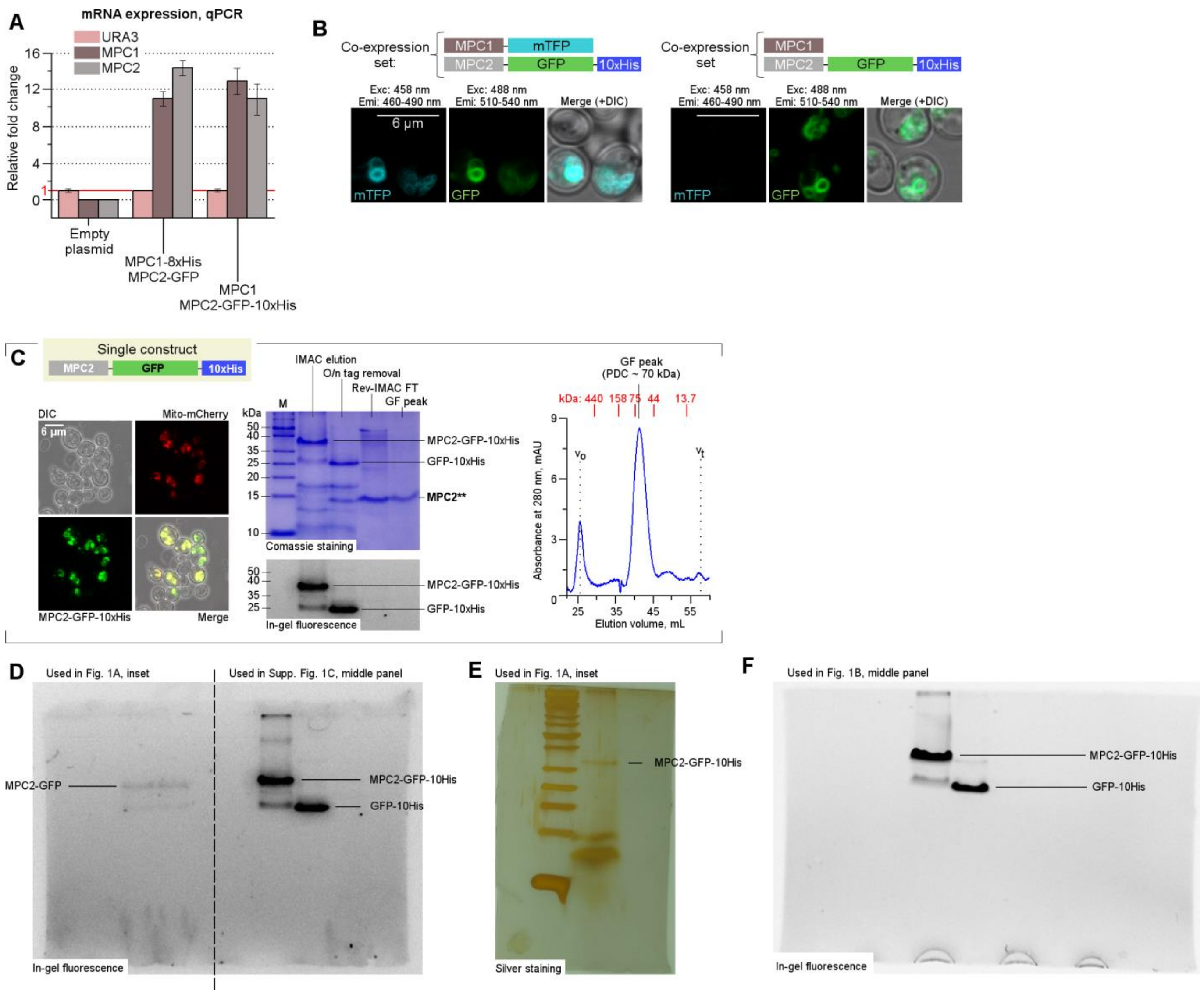

Recombinant co-expression of human MPC subunits in yeast. (A) In order to confirm the proper expression of the recombinant MPC subunits at the transcriptional level, and under control of galactose-inducible promoters GAL1 (for MPC1) and GAL10 (for MPC2), quantitative polymerase chain reaction (qPCR) was performed, upon mRNA extraction. Expression of actin was used a housekeeping marker and subsequent normalization by URA3 levels was performed. Values are given as expression fold change, relative to URA3 expression (red solid line). Values are mean ± s.d. (for quadruplicates). **(B)** Mitochondrial localization of MPC1 is confirmed, by the specific detection of a C-terminally fused mTFP (left panel), compared to unlabeled MPC1 (right panel). Respective excitation wavelengths and the emission detection windows for emission are described. **(C)** Diagram of the single expressed MPC2 construct and corresponding confocal microscopy (left panel). Middle panel: Electrophoretic (Tricine-SDS-PAGE)

analysis of representative chromatography steps. PP: PreScission Protease. Right panel: GF peak analysis revealed that pure monodisperse MPC2 (associated with DDM) was obtained at an equivalent molecular weight of 70 kDa. In the gel-filtration profile above, v_o indicates void volume, and v_t indicates the total liquid volume of the GF column. The corresponding elution volumes for calibration standards are shown in red. **(D)**, **(E)** and **(F)** Full-length gels from which silver-stained or fluorescent lanes in main text Fig. 1 and Supp. Fig. 1C were cropped.

Supplementary Figure 2.

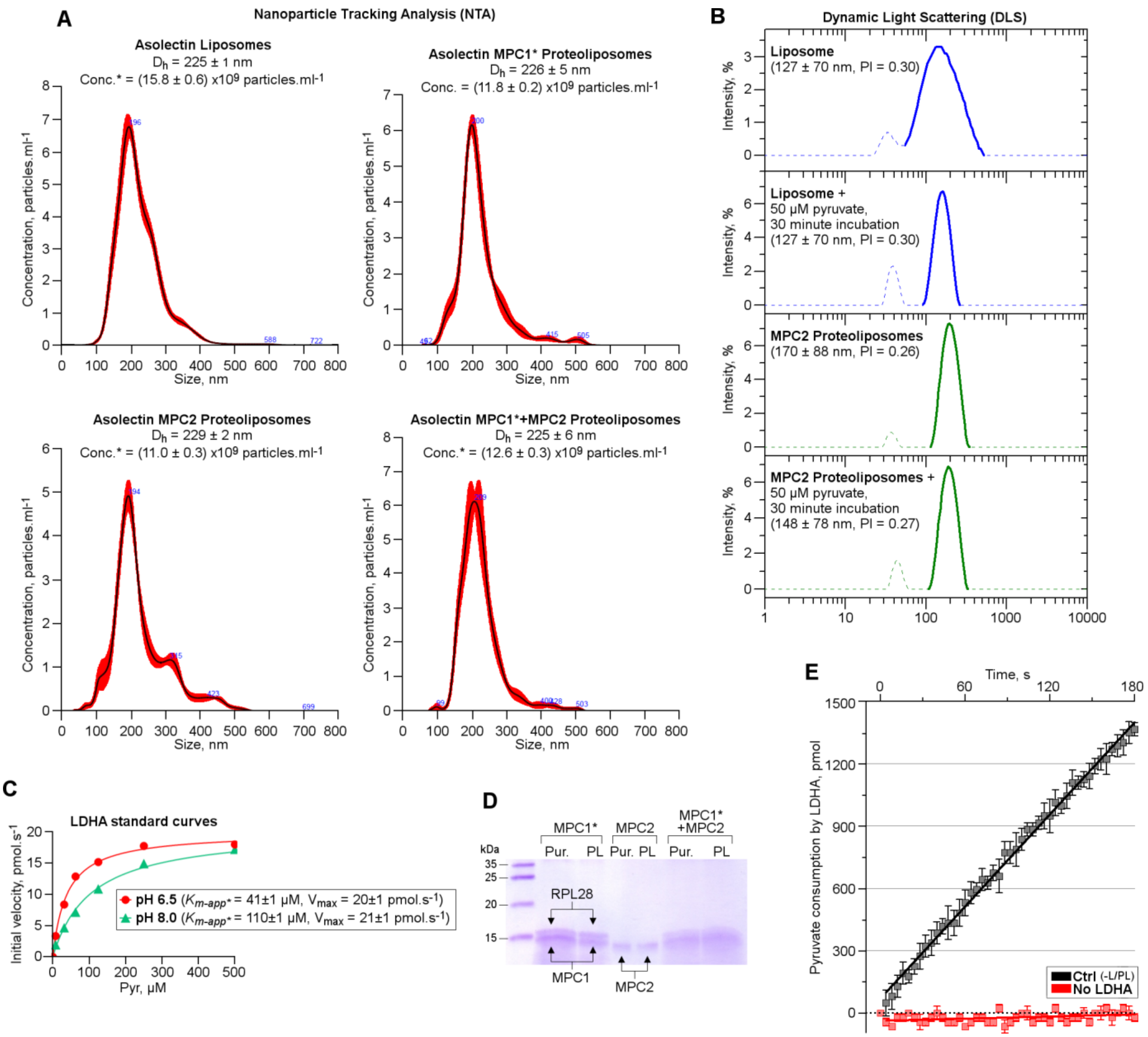

Qualitative analysis of lipo- and proteoliposomes, MPC1:MPC2 co-reconstitution and LDHA parametrization. (A) Size and concentration analysis of asolectin liposomes, MPC1*, MPC2 and MPC1*+MPC2 proteolipsomes using Nanoparticle tracking analysis (NTA). An asterisk (*) indicates that MPC1 was co-purified with yeast RPL28. **(B)** To check for undesired swelling or bursting during the transport assays, protein-free liposomes and MPC2 proteoliposomes size distributions were assessed by dynamic light scattering (DLS) for before and after 30min incubation with 50 µM pyruvate. **(C)** Standard kinetic curves for LDHA, from which Michaelian parameters were extracted from different conditions and respective hyperbolic fitting: pH 6.5 (red circles) and pH 8.0 (green triangles). The respective apparent

Km (Km-app, in respect to the final NADH concentration of 125 µM) and maximum velocities (Vmax) are indicated. **(D)** Electrophoretic analysis showing that equivalent amounts of individually purified (lanes labelled Pur.) MPC1* and MPC2 were mixed to produce co-reconstituted proteoliposomes (lanes labelled PL). An asterisk (*) indicates that MPC1 was co-purified with yeast RPL28. **(E)** Significant spontaneous oxidation of NADH (red), compared to the control activity of LDHA (black), is not observed during the timeframe of the experiments.

Supplementary Figure 3.

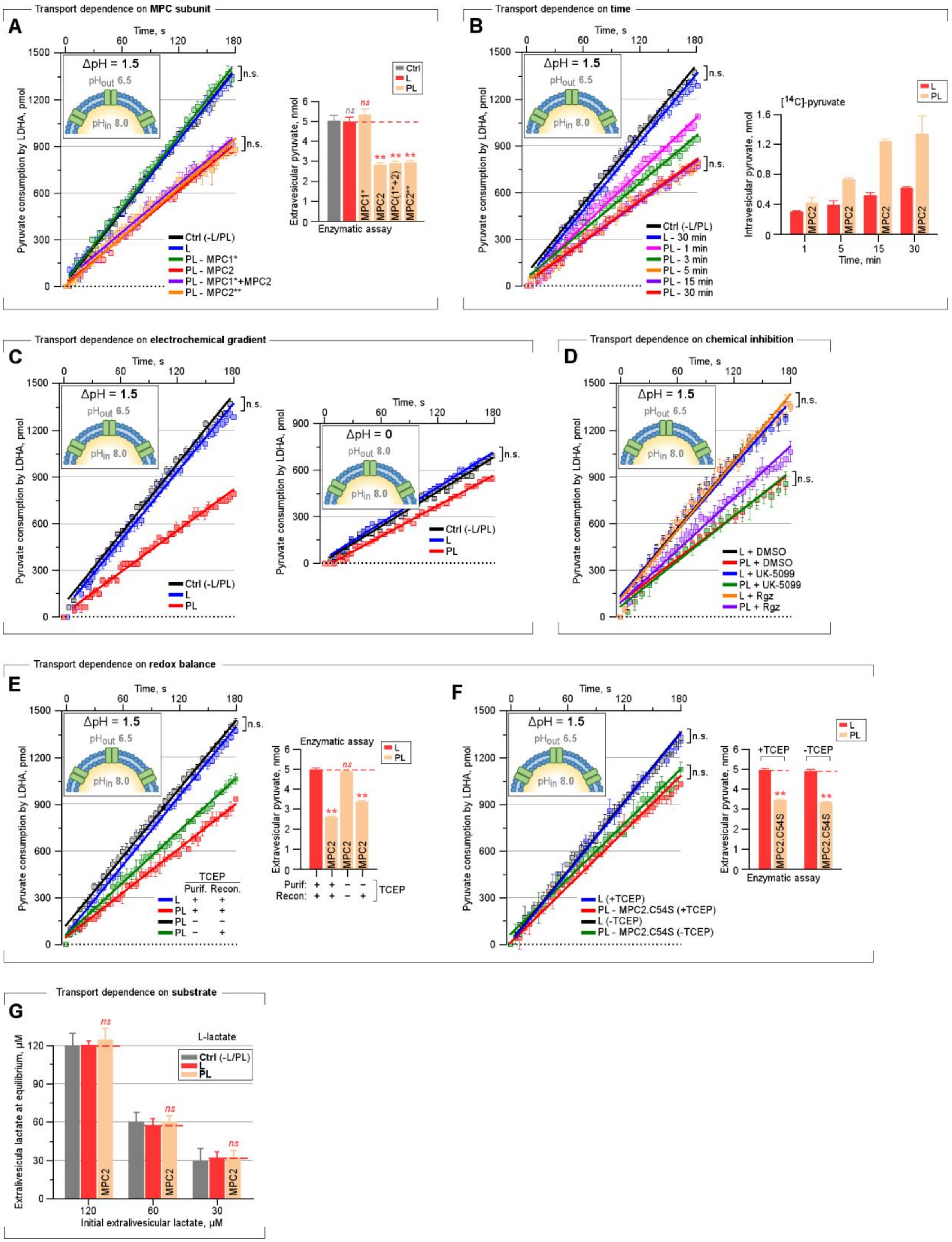

***In vitro* activity of human MPC. (A)** Quantification of extraliposomal pyruvate, as detected based on LDHA activity, in liposomes (L, blue) and proteoliposomes reconstituted with MPC1 and MPC2, individually (PL in green and red, respectively) and in combination (PL, violet). The inset indicates the pH gradient across the outer and inner vesicle environments. The control initial velocity for LDHA, at pH 6.5, in a liposome/proteoliposome-free condition is depicted as Ctrl (-L/PL), in black. An asterisk (*) indicates that MPC1 was co-purified with yeast RPL28. Two asterisk (**) indicate the activity of the MPC2 that was expressed and purified in the total absence of MPC1, as in Supporting Fig. 1C. **(B)** Quantification of external pyruvate in the liposome and MPC2-proteoliposome as a function of different incubation times (at a ΔpH of 1.5), as detected based on LDHA activity (left panel) and by using radiolabeled ^{14}C-pyruvate. **(C)** Quantification of external pyruvate for ΔpH = 1.5 units and ΔpH = 0t, as detected based on LDHA activity. **(D)** Quantification of external pyruvate for a collapsed pH gradient (as indicated in the inset), as detected based on LDHA activity. **(E)** Quantification of extraliposomal pyruvate in liposomes (L, blue) and proteoliposomes reconstituted with wild-type MPC2 in the presence (PL, red) and absence of TCEP (PL, black), as well as supplemented TCEP (PL, green). The inset indicates the pH gradient across the outer and inner vesicle environments. **(F)** Quantification of extraliposomal pyruvate in liposomes (L, blue) and proteoliposomes reconstituted with the isosteric MPC2 mutant (MPC2.C54S) in the presence (L, blue; PL, red) and absence of TCEP (L, black; PL, green). The red and green curves indicate that the activity of MPC2.C54S is independent of TCEP. In all cases above **(A-F)**, initial velocities for which the differences are statistically non-significant (n.s.; ANCOVA $P > 0.01$) are indicated.

Supplementary Figure 4.

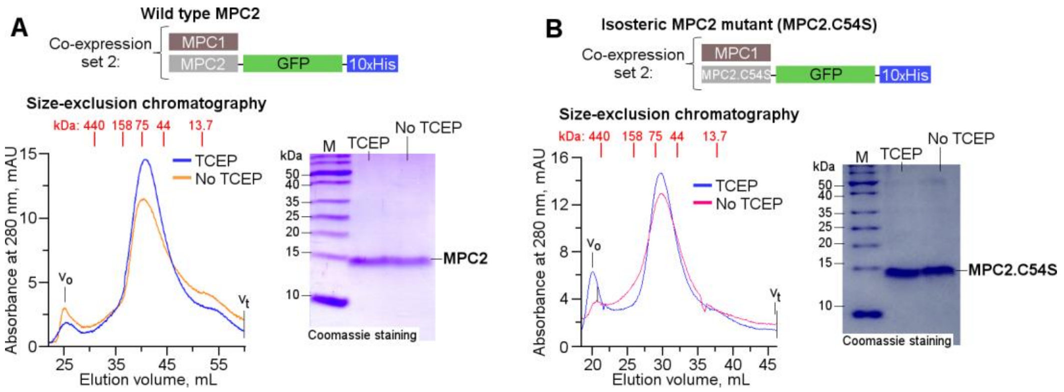

Purification of wild-type MPC2 in the absence of TCEP, and purification of the isosteric MPC2 mutant (MPC2.C54S). (A) A schematic depiction of human wild type MPC2-10xHis protein constructs co-expressed in yeast. Gel-filtration profile of wild type MPC2-10xHis in the presence (Blue) and absence TCEP (Orange). Peaks were obtained at an equivalent molecular weight of 70 kDa. v_o indicates void volume and v_t indicates the total liquid volume of the GF column. The corresponding elution volumes for calibration standards are shown in red. Inset represents the electrophoretic (tricine-SDS-PAGE) analysis of wild type MPC2-10xHis in the presence and absence of TCEP. The results enclosed in the right box depict the cinnamte independent transport activity of wildtype MPC2. **(B)** A schematic depiction of isosteric MPC2 mutant (MPC2.C54S) protein constructs co-expressed in yeast. Gel-filtration profile of isosteric MPC2 mutant (MPC2.C54S) in the presence (Blue) and absence TCEP (Pink). Peaks were obtained at an equivalent molecular weight of 70 kDa. v_o indicates void volume and v_t indicates the total liquid volume of the GF column. The corresponding elution volumes for calibration standards are shown in red. Inset represents the electrophoretic (tricine-SDS-PAGE) analysis of isosteric MPC2 mutant (MPC2.C54S) in the presence and absence of TC

Supplementary Figure 5.

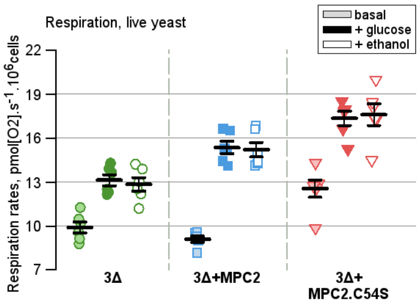

Cell respiration as function of the presence of MPC2. Oxygen consumption rates for 3Δ cells, displayed as basal rates (i.e., total absence of glucose, light colored shapes), after glucose addition (filled shapes) and after ethanol addition (empty shapes). Data for the control 3Δ cells transformed with empty pBEVY plasmid are shown as circles, and for 3Δ cells expressing either wild-type human MPC2 or the isosteric mutant (C54S) are shown as squares and triangles, respectively.

Supplementary Figure 6.

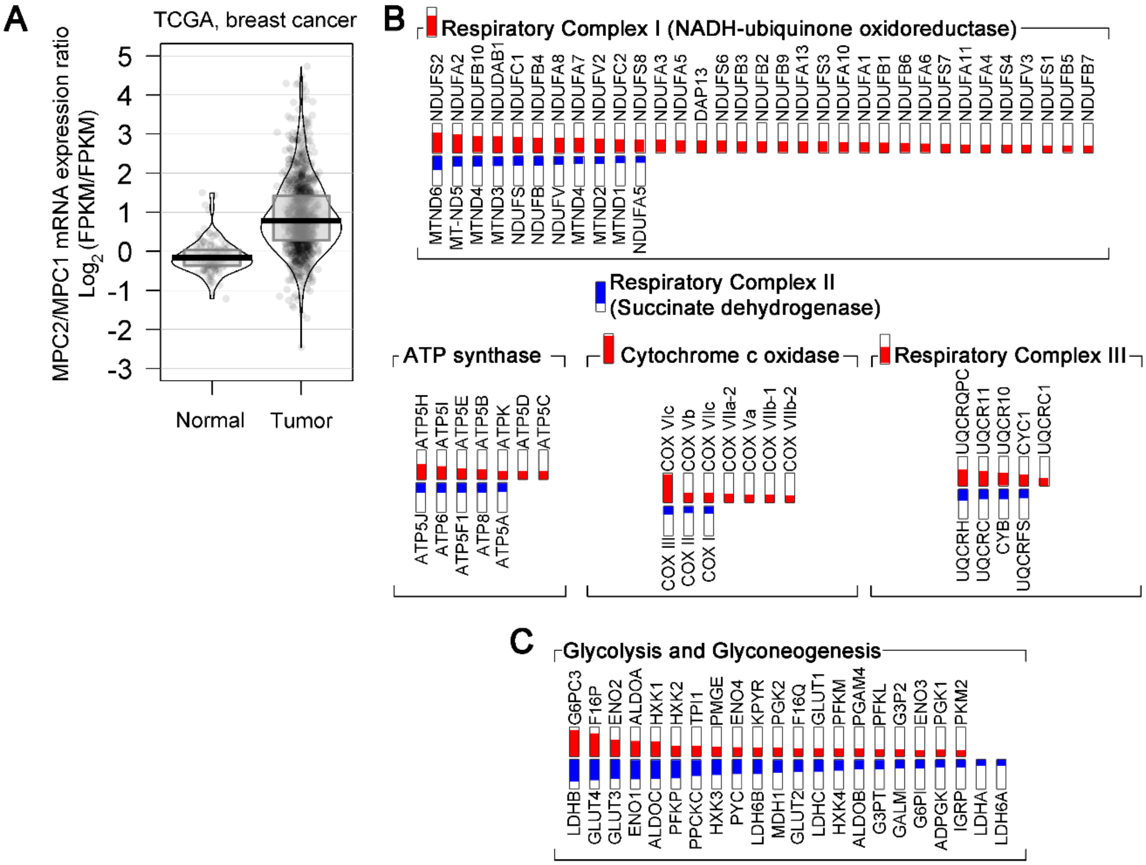

MPC2/MPC1 expression ration in breast cancer. (A) mRNA expression ratio for MPC2/MPC1 in normal and cancer cells according to the Breast Invasive Carcinoma (BRCA) dataset publicly available at The Cancer Genome Atlas project (Welch's t-test p-value < 2.2e-16). Positive (red meter) or negative (blue meter) correlation between the increased MPC2/MPC1 ratio and the expression of genes belonging to the (B) Oxidative Phosphorylation and (C) Glycolysis and Gluconeogenesis pathways, according to a Metacore analysis (Fisher's Exact Test two-tailed p-value = 0.0004)

,
